# Sparsity-based multi-height phase recovery in holographic microscopy

**DOI:** 10.1038/srep37862

**Published:** 2016-11-30

**Authors:** Yair Rivenson, Yichen Wu, Hongda Wang, Yibo Zhang, Alborz Feizi, Aydogan Ozcan

**Affiliations:** 1 Electrical Engineering Department, University of California, Los Angeles, CA, 90095, USA; 2 Bioengineering Department, University of California, Los Angeles, CA, 90095, USA; 3 California NanoSystems Institute (CNSI), University of California, Los Angeles, CA, 90095, USA; 4 Department of Surgery, David Geffen School of Medicine, University of California, Los Angeles, CA, 90095, USA

## Abstract

High-resolution imaging of densely connected samples such as pathology slides using digital in-line holographic microscopy requires the acquisition of several holograms, e.g., at >6–8 different sample-to-sensor distances, to achieve robust phase recovery and coherent imaging of specimen. Reducing the number of these holographic measurements would normally result in reconstruction artifacts and loss of image quality, which would be detrimental especially for biomedical and diagnostics-related applications. Inspired by the fact that most natural images are sparse in some domain, here we introduce a sparsity-based phase reconstruction technique implemented in wavelet domain to achieve at least 2-fold reduction in the number of holographic measurements for coherent imaging of densely connected samples with minimal impact on the reconstructed image quality, quantified using a structural similarity index. We demonstrated the success of this approach by imaging Papanicolaou smears and breast cancer tissue slides over a large field-of-view of ~20 mm^2^ using 2 in-line holograms that are acquired at different sample-to-sensor distances and processed using sparsity-based multi-height phase recovery. This new phase recovery approach that makes use of sparsity can also be extended to other coherent imaging schemes, involving e.g., multiple illumination angles or wavelengths to increase the throughput and speed of coherent imaging.

Lensfree digital in-line holographic microscopy^1^,^2^ is a rapidly emerging computational imaging technique, which allows highly compact and high-throughput microscope designs. It is enabled by leveraging constant advances and improvements in microscopy and image reconstruction techniques as well as image sensor technology and computational power, mostly driven by consumer electronics industry. Its current implementations can achieve gigapixel level space-bandwidth-products, by employing cost-effective and field-portable imaging hardware^1^,^2^,^3^,^4^. In order to keep the imaging setup as compact as possible, the on-chip holographic image acquisition platform employs an in-line holography geometry^5^, where the scattered object field and the un-scattered reference beam co-propagate in the same direction, and the intensity of the interference pattern between these two beams is recorded by a digital image sensor-array. Because the recorded hologram only contains the intensity information of the complex optical field, direct back-propagation of this in-line hologram to the object plane will generate a spatial artifact called twin image on top of the object’s original image. Unlike an off-axis holographic imaging geometry^5^, where the twin image artifact can be robustly removed by angled wave propagation, in-line holography is more susceptible to this twin image related artifact term^5^. The negative impact of this artifact on image quality is further amplified owing to the small sample-to-sensor distances that are used in on-chip implementations of digital in-line holographic microscopy, where the sample field-of-view is equal to the sensor active area^2^,^3^,^6^,^7^,^8^,^9^,^10^.

Twin image artifact in digital in-line holography can also be computationally eliminated by imposing physical constraints that the twin image does not satisfy. Based on such constraints, a twin-image-free object can be retrieved through e.g., an iterative error reduction algorithm^7^,^8^. One of the earliest explored constraints for this purpose is the object support where a threshold defines a 2D object mask and the back-propagated field on the object plane outside the mask is considered as noise and iteratively removed^1^,^8^. Although this simple approach requires a single hologram measurement, this constraint works better for relatively isolated objects and its implementation is challenging in dealing with dense and connected samples, such as pathology slides, which are of significant importance in biomedical diagnosis.

To address this phase retrieval problem^5^,^7^,^8^ of in-line holography it is common to apply measurement diversity which can include, e.g. sample-to-sensor distances^9^,^10^,^11^,^12^, illumination angles^13^,^14^ and wavelengths^14^,^15^. However, previous efforts have shown that for imaging of spatially connected and dense biological objects such as pathology slides and tissue samples, the measurements always need to be *oversampled* in one domain, with several additional images acquired with different physical parameters. For instance, in imaging pathology slides using multi-height measurements, usually 6–8 holograms at different sample-to-sensor distances are required to get high quality and clinically relevant microscopic reconstructions (amplitude and phase images) of the object^10^, i.e., the number of measurements is 3–4 times of the number of variables, including the amplitude *and* phase pixels that needed to be retrieved in a complex image of the sample. This increase in the number of measurements also increases the data acquisition and processing times, limiting the throughput of the imaging system.

Inspired by the fact that the images of most natural objects, such as biological specimen, can be sparsely represented in some wavelet domain^16^, here we introduce the use of a sparsity constraint in the wavelet domain, improving multi-height based phase retrieval, to significantly reduce the required number of measurements while maintaining the quality of the reconstructed phase and amplitude images of the objects. We experimentally demonstrate that for densely connected biological samples, such as Papanicolaou smears and breast cancer tissue slides, 2 in-line holograms with different sample-to-sensor distances are sufficient for image reconstruction, when sparsity constraints are applied during the iterative reconstruction process. The resulting reconstructed object image quality is comparable to the ones that can be reconstructed from >4–8 different measurements using conventional multi-height phase recovery methods^2^,^9^,^10^. This means, by making use of a sparsity constraint in our reconstruction, we achieved at least 2-fold decrease in the number of holograms that need to be acquired. Furthermore, if we consider the number of unknown variables as 2 *N* (i.e., the amplitude and phase images of the object, each with *N* pixels), the number of measurements is also 2 *N* in this sparsity-based multi-height phase recovery approach, which means ***the physical measurement space is no longer oversampled unlike the previous multi-height phase and image recovery approaches***. In fact, the additional sparsity constraint in wavelet domain enables us to go below a 3*N*-2 measurement limit that has been previously shown to be the smallest number of measurements needed for robust phase retrieval^17^,^18^.

Note that the sparsity constraint in our approach is quite different from the previously reported compressive holography efforts^19^,^20^,^21^,^22^. These earlier reports imaged isolated objects and have shown that the free-space propagation in itself is an extremely efficient encoding mechanism for compressive sensing, allowing the inference of higher dimensional data from traditionally undersampled projections^23^,^24^,^25^. Here, we demonstrate the ability of wavelet domain sparsity encoding for multi-height-based phase recovery and demonstrate its success for clinically relevant dense samples, including highly connected pathology slides of breast tissue and Papanicolaou (Pap) smears that are imaged over a large field-of-view of ~20 mm^2^, equal to the active area of our image sensor chip. In fact, as a result of this significant difference in the density and connectivity of the object to be imaged, the number of measurements that we need to have without losing image quality is two, rather than a single hologram.

This sparsity-based phase recovery approach can also be extended to other coherent microscopy schemes, involving e.g., multi-angle^13^ or multi-wavelength-based^26^ phase retrieval. Furthermore, this technique can be potentially combined with a recently introduced phasor approach for high resolution and wide field-of-view imaging^27^ and/or multiplexed color imaging^28^ to further reduce the number of measurements in these holographic microscopy approaches. Enabled by novel algorithmic processing, this sparsity-based holographic image reconstruction technique can be regarded as another step forward in making lensfree on-chip holography more efficient, higher throughput and more appealing in microscopy-related applications.

## Methods

### Sparse object representation

Image recovery based on sparsity constraints is a paradigm which has been applied for many different imaging-related tasks such as denoising, inpainting, deblurring, compression and compressed sensing/sampling^29^. In this framework, we wish to recover the discrete approximation of a complex sample, 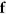, where 2 *N* is the total number of pixels which is required to represent this complex-valued sample/object with independent phase and amplitude channels, each having *N* pixels^5^. The main assumption of sparse image recovery is that the sought signal can be written as a linear combination of a small number of basis elements, 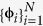, such that for 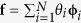, the number of significant coefficients of **θ** = (*θ*_1_, .., *θ*_*N*_), which are required for accurate signal representation, *S*, is much less than *N*, i.e., *S* ≪ *N*. For dense biological samples including tissue sections and smears that we used in this manuscript, we found that the reconstructed coherent imaging results acquired by using 8 different sample-to-sensor distances (which we consider as our clinically relevant reference standard as confirmed in an earlier study^10^) can be accurately represented using a very small *S*, i.e., *ρ* = *S*/*N* ≈ 0.07:0.15 through a mathematical transformation such as CDF 9/7 wavelet transform^30^,^31^ which is one of the leading non-adaptive image compression techniques, also applied in JPEG-2000 image compression standard. This observation is used as a *loose* constraint for the number of sparse coefficients to be utilized during our iterative object reconstruction process, which will be detailed below.

Another dimension of sparsity-based image reconstruction involves nonlinear operators which can be performed on the signal. One of the common sparsity promoting operators which is used in imaging-related applications is known as the total variation norm^32^, i.e., *TV*(**f**) which quantifies the magnitude of the gradient of the signal:


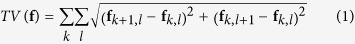


where *k* and *l* are pixel indexes in the reconstructed image. This operator has been shown to be extremely useful in image processing tasks such as denoising, deblurring and compressed sensing, specifically with holographically acquired data^19^,^20^,^33^,^34^. A total variation norm based constraint aims the preservation of sharp boundaries of the object with smooth spatial textures confined between them. In this work, we apply the total variation operator in the wavelet domain in order to suppress noise within our iterative reconstruction process, which will be detailed below.

### Lensfree on chip imaging setup

**Figure 1:**
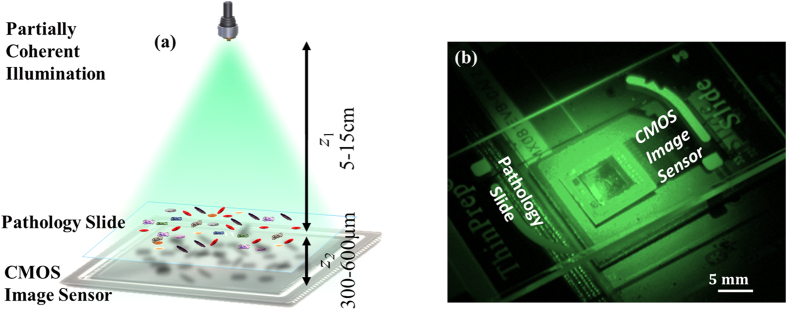
(**a**) Schematics and (**b**) picture of our lensfree holographic on-chip microscopy setup.

A schematic figure of our lensfree holographic on-chip microscope is shown in Fig. 1. A broadband illumination source (WhiteLase micro, Fianium Ltd.) is filtered by an acousto-optic tunable filter to output partially coherent light within the visible spectrum with a spectral bandwidth of ~2–3 nm. The light is coupled into a single-mode optical fiber, and the emitted light from the fiber tip propagates a distance of ~5–15 cm before impinging on the sample plane, which is mounted on a 3D-printed sample holder. The sample is placed ~300–600 μm above the active area of a CMOS image sensor chip (IMX081, Sony, 1.12 μm pixel size, 16.4 Megapixels). In this on-chip imaging configuration, the sample field-of-view is equal to the sensor chip active area, i.e., ~20 mm^2^. The image sensor is attached to a positioning stage (MAX606, Thorlabs, Inc.), which is used for alignment, image sensor translation (to perform pixel super-resolution) and acquisition of several holograms with different sample-to-sensor distances, 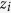. Acquisition of several images with different sample-to-sensor distances generates a series of measurement constraints which are used for multi-height-based phase recovery, detailed in the next sub-sections. A custom developed LabVIEW program coordinates different components of this setup during the entire image acquisition stage.

### Hologram acquisition and pre-processing

In our experiments, a series of wide field-of-view and low resolution (1.12 μm pixel size, before the pixel super-resolution step) holograms were acquired at each sensor-to-sample distance. For a given illumination wavelength, λ, refractive index, *n,* and sample-to-sensor distance 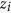 the hologram formation at the sensor plane can be written as:


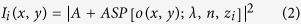


where *o* is the complex-valued object function, *A* is the amplitude of the reference (plane) wave and the 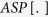 operator is the *angular spectrum based free-space propagation* of the illuminated object, which can be calculated by the spatial Fourier transform of the input signal and then multiplying it by the following filter (defined over the spatial frequency variables, υ_*x*_, υ_*y*_):


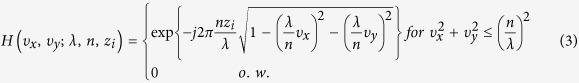


which is then followed by an inverse 2D Fourier transform. The hologram intensity, 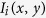, is sampled by the imaging sensor chip with a sampling interval which corresponds to the pixel pitch. In order to generate higher resolution holograms, the stage that holds the sensor chip was programmed to move the sensor laterally on a 6 ×6 grid and at each grid point a lower resolution hologram was acquired. Applying a conjugate-gradient-based pixel super-resolution method^35^,^36^ on these 6 × 6 holograms results in a new hologram with an effective pixel size of ~0.37 μm. Next, the process is repeated for 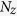 different sensor-to-sample distances, i.e., 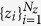 in order to create the measurement diversity required for standard phase recovery. Following the hologram acquisition, these super-resolved holograms are digitally aligned with respect to each other and the estimated sample-to-sensor distance is refined using an auto-focusing algorithm^37^.

### Initial phase estimation using the Transport-of-Intensity Equation (TIE)

Previous reports^10^ have demonstrated that the effectiveness of the image reconstruction process using lensfree multi-height holograms can be substantially accelerated by solving the TIE^38^,^39^,^40^ to obtain an initial phase guess. The TIE is a deterministic phase retrieval method that generates the solution from a set of two or more diffraction patterns or holograms, which are acquired at different sample-to-sensor distances. Unfortunately, this analytical solution is a lower-resolution method and cannot in itself generate a high-resolution object reconstruction in lensfree on-chip microscopy, and that is why it is followed by an iterative phase refinement method, as detailed below.

### Iterative multi-height phase recovery

The multi-height-based phase recovery approach^9^,^10^,^41^ is an iterative error-reduction algorithm which uses the acquired holograms at various sample-to-sensor distances as a set of physical constraints to correct the estimated phase in each iteration. In this algorithm, the lower-resolution phase result obtained by the TIE method is used as the initial phase term to accompany the field amplitude that is acquired at the plane that is closest to the sensor chip. This newly formed complex field is then numerically propagated to the plane of the next sample-to-sensor distance, where its amplitude is averaged with the square root of the second hologram, and the phase is retained for the next step of the iteration. The same procedure is repeated for all the other acquired holograms at different sample-to-sensor distances and then the process is repeated in a reverse fashion, i.e., from larger sample-to-sensor distances toward smaller, all the way to the first plane that is the closest to the sensor chip. This iterative algorithm is terminated after typically ~10–30 iterations or once a convergence criterion has been achieved. Following the termination of the iterative process, the refined complex field at the plane closest to the sensor plane is numerically back-propagated to the object plane. This complex-valued result,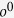, is used as the initial object guess and fed to the sparsity constrained reconstruction algorithm which will be discussed in the following sub-section.

### Sparsity-based multi-height phase recovery

**Figure 2:**
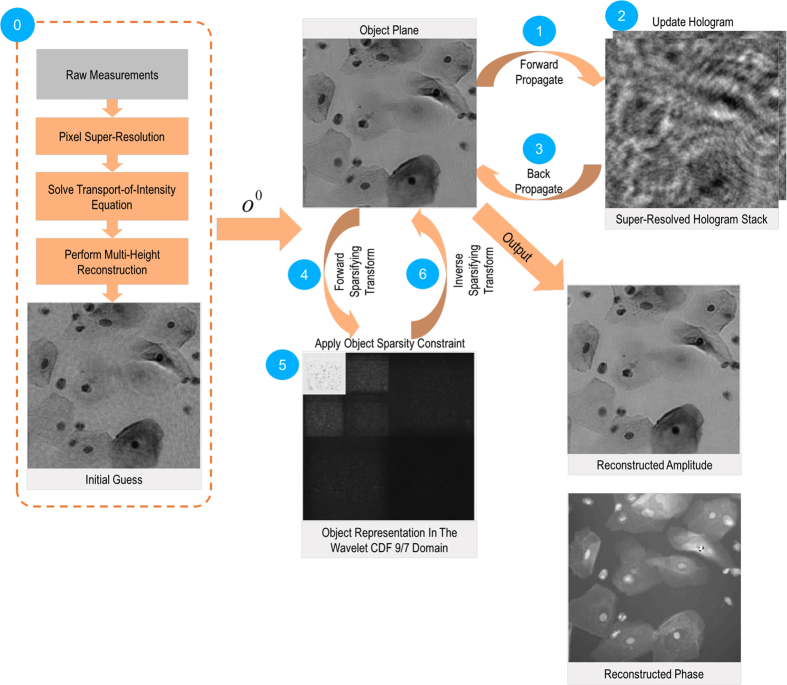
Schematic diagram of sparsity-based multi-height phase recovery algorithm. After ~100 iterations or the fulfilment of the convergence condition (if earlier), amplitude and phase images of the sample are reconstructed.

Sparsity-based multi-height phase recovery algorithm, as summarized in the right panel of Fig. 2, can be described as follows:

*Step 1* – Perform numerical forward propagation of the current guess, 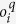 to the *i*-th hologram plane, 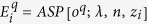, with 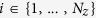.

*Step 2* - Update the magnitude according to: 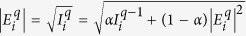 and keep the phase of 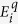. Typical range of values for our update parameter is 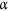
~0.5–0.9.

*Step 3* - Perform backward angular spectrum propagation for the updated complex field amplitude, 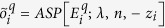.

*Step 4* – Project the object function to the sparsifying (Wavelet) domain, which results in a coefficient set 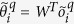, where (.)^*T*^ refers to the transpose operation.

*Step 5* - Apply the object sparsity constraint to the result of Step 4:

- Update the sparse support area for the *magnitude*, by keeping the largest *S* coefficients and update the remaining (*N*-*S*) coefficients. To achieve this, we first define the sparsity support region (*Λ*) which contains the most significant *S* coefficients within 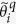.
- Keep all the elements within 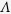 unchanged, i.e., 
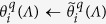
.
- Reduce the error *outside* of the sparse support region in the Wavelet domain, which is defined by *Λ*^*C*^, by performing the following iterative update: 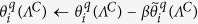, where *β* is a relaxation coefficient, e.g., *β*~0.7–0.9.
- Perform total variation denoising in the Wavelet domain to achieve two aims: (*i*) Smoothen out the regions where the low frequency components of the twin image are more dominant; and (*ii*) preserve the edges (details) of the objects in the higher frequencies of the image, helping to reduce the measurement noise as well as self-interference related terms. The total variation denoising algorithm can be implemented by using either the original formulation of Rudin-Osher-Fatemi or Chambolle’s algorithm. We used:

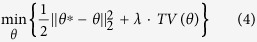


where 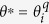 from Step 5(c), *θ* is the variable of the denoising algorithm, *TV(θ*) is the total-variation norm, which is defined in Equation (1) and 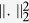 is the *l*2-norm, which serves as a fidelity term. The parameter *λ* is a tuning parameter, which can be adaptively refined^32^ or selected *a priori* and it controls the tradeoff between data fidelity and denoising. Generally, we perform ~1–2 iterations in order not to introduce spatial blurring to the reconstruction. Also, since large values of *λ* favors blurring, it should be carefully chosen^43^ and we typically set *λ* to be ~0.002:0.01 for intensity normalized biological samples.

*Step 6* - Update the object estimate by applying an inverse wavelet transform on the solution of Equation (4), 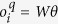, to return to the object space for the next iteration.

Following Step 6, Step 1 is repeated for all the *N*_*z*_ acquired holograms at different sample-to-sensor distances. Following the update of the solution, 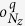 and the magnitude corresponding to the hologram measured at *N*_*z*_-th plane, we proceed to the next iteration by incrementing 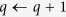. This algorithm is repeated for ~100 iterations, or until a convergence criterion is met, for example when smaller than a predefined update tolerance between two consecutive iterations is achieved in either the object or hologram planes.

The entire reconstruction algorithm is implemented using Matlab on a computer with an Intel Xeon E5–2667v3 3.2 GHz CPU (central processing unit) and 256 GB of RAM (Random Access Memory), running Windows Server R2012 R2 operating system. For a field-of-view of 1.1 × 1.1 mm^2^, the entire reconstruction process took ~28 minutes for *N*_*z*_ = 2 and 6 × 6 = 36 raw holograms for pixel super-resolution at each height. Implementation of the presented algorithm using a dedicated parallel computing platform and programming environment on a GPU (Graphics Processing Unit) should yield a significant speed improvement^44^.

### Evaluation of the image reconstruction quality

Our image reconstruction quality assessment is based on (*i*) visual inspection of the results in comparison to our clinically relevant reference coherent images^10^ and (*ii*) *quantitatively* applying structural similarity index (SSIM)^45^ on the reconstructed object image. The SSIM has been proven to be more consistent with the human visual system when compared to peak signal-to-noise ratio (PSNR) and mean square-error (MSE)^45^ based image evaluation criteria. The SSIM quantifies the changes in structural information by inspecting the relationship among the image contrast, luminance, and structure components. The contrast is evaluated as the standard deviation of an image:


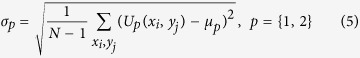


where *μ*_*p*_ is the luminance (mean) of the *p*-th image, *U*_*p*_. The structural measurement is estimated using the cross-covariance between two images that are compared to each other:


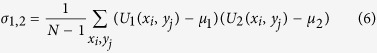


Based on these definitions, the SSIM between two images is given by:


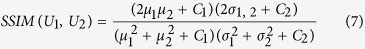


where *C*_1_, *C*_2_ are stabilization constants, which prevent division by a small denominator. These coefficients^45^ are selected as: *C*_1_ = (*K*_1_*L*)^2^ and *C*_2_ = (*K*_2_*L*)^2^ with *K*_1_, *K*_2_ ≪ 1 and *L* is the dynamic range of the image, e.g., 255 for an 8-bit grayscale image.

### Sample Preparation

De-identified Pap smear slide was provided by UCLA Department of Pathology (Institutional Review Board no. 11–003335) using ThinPrep® preparation. De-identified Hematoxylin and Eosin (H&E) stained human breast cancer tissue slides were acquired from the UCLA Translational Pathology Core Laboratory. We used existing and anonymous specimen, where no subject related information is linked or can be retrieved.

## Results and Discussion

**Figure 3:**
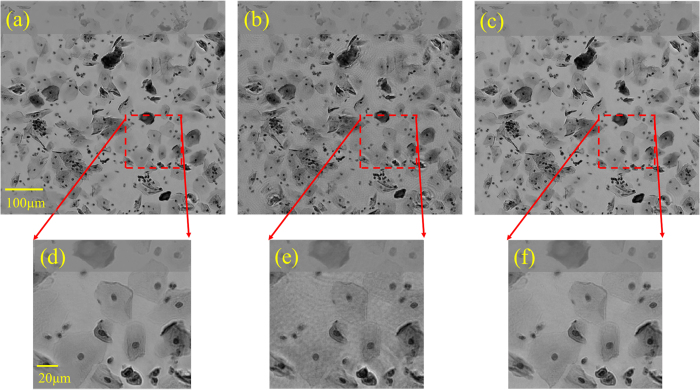
Comparison of reconstruction results corresponding to a Pap smear sample. Left column: the reconstruction result that is obtained by using *N_z_ *= 8 holograms captured at different sensor-to-sample distances processed by the standard multi-height phase retrieval method, which serves as our reference image. Middle column: Same as the left column, except for *N_z_* = 2. Right column: Reconstruction result using the same *N_z_* = 2 acquired holograms, processed by our sparsity-based multi-height phase recovery algorithm, showing an excellent match to *N_z_* = 8 case shown in (**a,d**).

**Figure 4:**
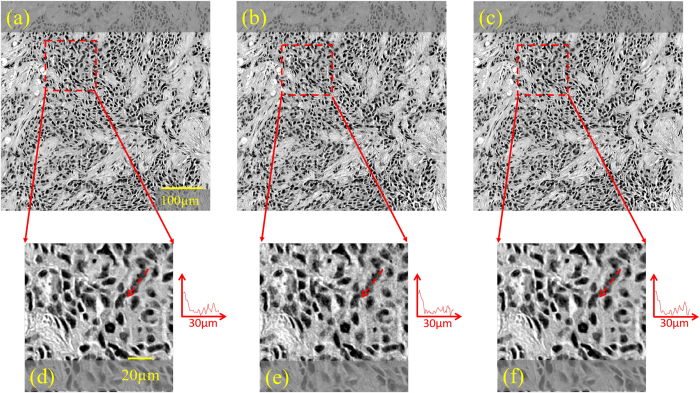
Same as Fig. 3, except for an H&E stained breast cancer pathology slide (~3 μm thick). A 30 μm length cross-section of a selected region of interest is also shown to the left of each zoomed-in image to assist in comparison of the reconstructed images.

In order to experimentally test the proposed sparsity-based image reconstruction algorithm, we acquired a set of 8 super-resolved holograms at different sample-to-sensor distances (~300–600 μm) corresponding to stained Papanicolaou (Pap) smears as well as H&E stained breast cancer tissue slides. First, we applied the multi-height based iterative phase recovery algorithm on all of these *N*_z_ = 8 pixel super-resolved holograms in order to get clinically relevant^10^ baseline reference images, which are shown in Figs 3(a,d) and 4(a,d). Once we attempt to reconstruct the images of the same samples using *N*_z_ = 2 holograms acquired at different sample-to-sensor distances with the same iterative multi-height phase retrieval algorithm, spatial artifacts appear which are illustrated in Figs 3(b,e) and 4(b,e). However, using the same 2 holograms with the proposed sparsity constrained multi-height phase retrieval algorithm, the reconstruction results, shown in Figs 3(c,f) and 4(c,f), significantly improve and become comparable in image quality to the reference images. In order to quantify our reconstruction quality, we also calculated the SSIM values for these images, with the results summarized in Table 1. For the Pap smear sample, the standard multi-height reconstruction of *N*_*z*_ = 2 pixel super-resolved holograms gave an SSIM value of 0.66, while the sparsity-based reconstruction using the same measurements gave an improved SSIM value of 0.89. Similarly, for the H&E stained breast cancer pathology slide, the SSIM value for the multi-height reconstruction (*N*_*z*_ = 2) was 0.73, while for the sparsity-based reconstruction the SSIM value increased to 0.83. Similar improvements in SSIM values using sparsity-based multi-height phase recovery were also observed for *N*_*z*_ = 4, as shown in Table 1. These results illustrate that we are gaining at least 2-fold imaging speed improvement with reduced number of measurements compared to the standard multi-height phase recovery approach, without compromising spatial resolution or the field-of-view of our on-chip microscope. This, in-turn, reduces the data bandwidth and storage related requirements, which is especially important for field-portable implementations of lensfree microscopy tools.

The presented sparsity-based multi-height phase retrieval method could also work without using pixel super-resolution. Nevertheless, the usage of and the need for pixel super-resolution depend on the targeted resolution in the reconstructed image. In this work, we used a CMOS image sensor that has a pixel size of 1.12 μm and to achieve a resolution comparable to a conventional benchtop microscope with e.g., a 40X objective lens we used the pixel super-resolution framework to digitally create effectively smaller pixels. As an alternative to lateral shifts between the hologram and the sensor array planes (which can be achieved by e.g., source shifting, multi-aperture illumination, sample shifting or sensor shifting), wavelength scanning^26^ over a narrow bandwidth (e.g., 10–30 nm) can also be used for rapid implementation of pixel super-resolution, which also has the advantage of creating a uniform resolution improvement across all the directions on the sample plane.

While the presented approach has been demonstrated for multi-height holographic imaging and phase recovery, other types of physical measurement diversities can also be utilized in the same sparse signal recovery framework, such as multi-angle illumination and wavelength scanning^26^,^27^ which might benefit various applications in quantitative imaging of live biological samples, such as growing colonies of bacteria, fungi or other types of cells.

It is also important to note that, in addition to the CDF 9/7 wavelet transform that we used in this work, other wavelet transforms can also be used in the same image reconstruction method. As described in the Methods Section, an effective way of doing this can be by applying several wavelet transforms on a known database of pre-acquired images, thus finding the best representation and getting a good approximation of the number of required coefficients for sparse signal recovery. Adaptive methods, such as dictionary learning^46^ and optimal basis generation^47^, which may yield over-complete linear signal representations could also be considered for the same framework^48^.

Since one of the main goals of this work is to use less number of raw measurements, while also preserving image quality, it is important to choose our measurements in a way that will help us converge to the correct result. Of great importance for a successful reconstruction is a careful initialization of the algorithm. Specifically, it has been shown that when the measurement operator is given by free-space propagation, special emphasis needs to be put on the low spatial frequencies^20^, which contain most of the information about the sample, and therefore the low frequency wavelet bands cannot be considered sparse. Since the low frequency phase curvature changes slowly as a function of the sample-to-sensor distance^39^, the distance between the first two measurements should be large enough to acquire changes in these low frequencies. However, the distance should not be too large, since the signal-to-noise-ratio (SNR) also decreases proportional to the sample-to-sensor distance. Practically, we found that an axial distance of ~100–150 μm between the two holographic measurements gives the best result for our initialization. On the other hand, to better resolve high spatial frequencies, we should choose a small sample-to-sensor distance, typically ~300 μm for our setup. The closer the acquired hologram, the more suitable it is to sense sparse frequency components^24^, while as we take our measurements further away from the object, the reconstruction would favor sparse objects, such as point sources and scatterers.

## Conclusions

We developed a sparsity-based phase reconstruction algorithm for digital in-line holographic imaging of densely connected samples. This algorithm is capable of reconstructing amplitude and phase images of biological samples using only 2 holograms acquired at different sample-to-sensor distances, which is at least 2-fold less compared to the number of holograms that is utilized in previous multi-height phase retrieval approaches. Stated differently, using this sparsity-based holographic phase retrieval method, we demonstrated that the number of the reconstructed pixels (i.e., 2*N,* including the phase and amplitude channels of the sample) can be made equal to the number of measured intensity-only pixels. We demonstrated the success of this approach by imaging Papanicolaou smears and breast cancer tissue slides over a large field-of-view of ~20 mm^2^. This sparsity-based phase retrieval method is also applicable to other high resolution holographic imaging techniques involving e.g., multiple illumination angles or wavelengths, both of which can be used for enhancing the space-bandwidth product of a coherent holographic microscope.

## Additional Information

**How to cite this article**: Rivenson, Y. *et al*. Sparsity-based multi-height phase recovery in holographic microscopy. *Sci. Rep.* **6**, 37862; doi: 10.1038/srep37862 (2016).

**Publisher's note:** Springer Nature remains neutral with regard to jurisdictional claims in published maps and institutional affiliations.

## Figures and Tables

**Table 1:** Summary of structural similarity index (SSIM) results for various reconstruction methods corresponding to Pap smear samples and breast tissue histopathology slides.

Reconstruction Method Sample	MH Phase-Recovery (*N*_*z*_ = 2)	Sparsity-based MH Phase-Recovery (*N*_*z*_ = 2)	MH Phase-Recovery (*N*_*z*_ = 4)	Sparsity-based MH Phase-Recovery (*N*_*z*_ = 4)	MH Phase-Recovery (*N*_*z*_ = 8)
Papanicolaou (Pap) Smear	0.66	0.89	0.9	0.94	1
H&E Stained Breast Tissue	0.73	0.83	0.83	0.87	1
